# Supplementary material for: Multi‐season analysis reveals hundreds of drought‐responsive genes in sorghum
Source: Plant J. 2026 Feb 2;125(3):e70657. doi: 10.1111/tpj.70657 (PMC12863931; doi:10.1111/tpj.70657)
Supplement: Supplementary file 1 — Figure S1. Variation between datasets. Figure S2. Pairwise comparison of log‐fold change in gene expression by year. Figure S3. DE genes recovered from each condition per year. Figure S4. Root expression of AMF‐related genes during drought. Figure S5. Expression of 7 pangenes corresponding to glyoxylate pathway loci in sorghum. Figure S6. Identification of stay‐green candidates in EPICON data. Figure S7. Comparison to meta‐analysis of sorghum drought transcriptomes. [file TPJ-125-0-s002.docx]

| **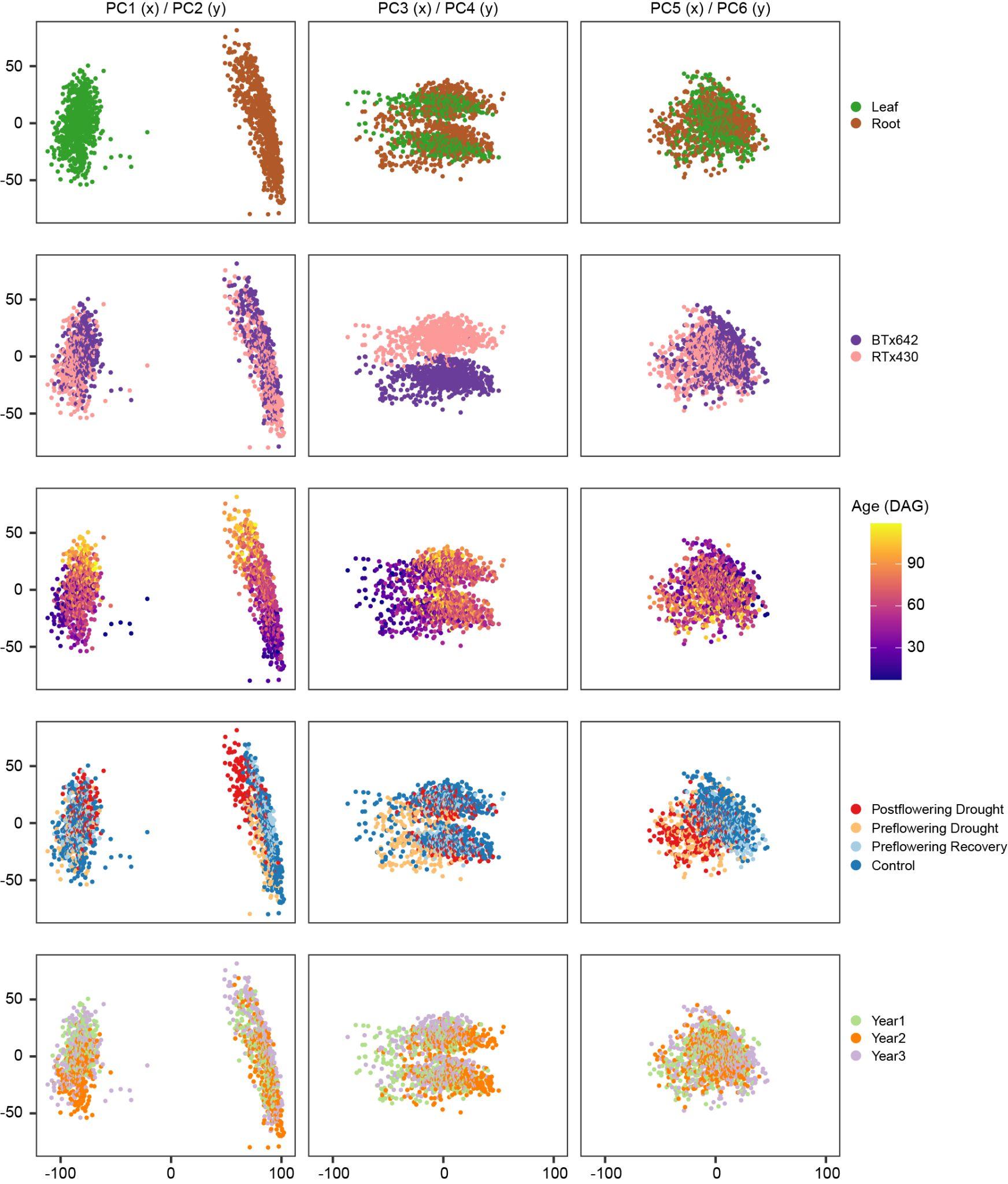** |
| --- |
| **Figure S1**: Variation between datasets. PCA was conducted with all transcriptomes, with the first six principal components plotted. |

| **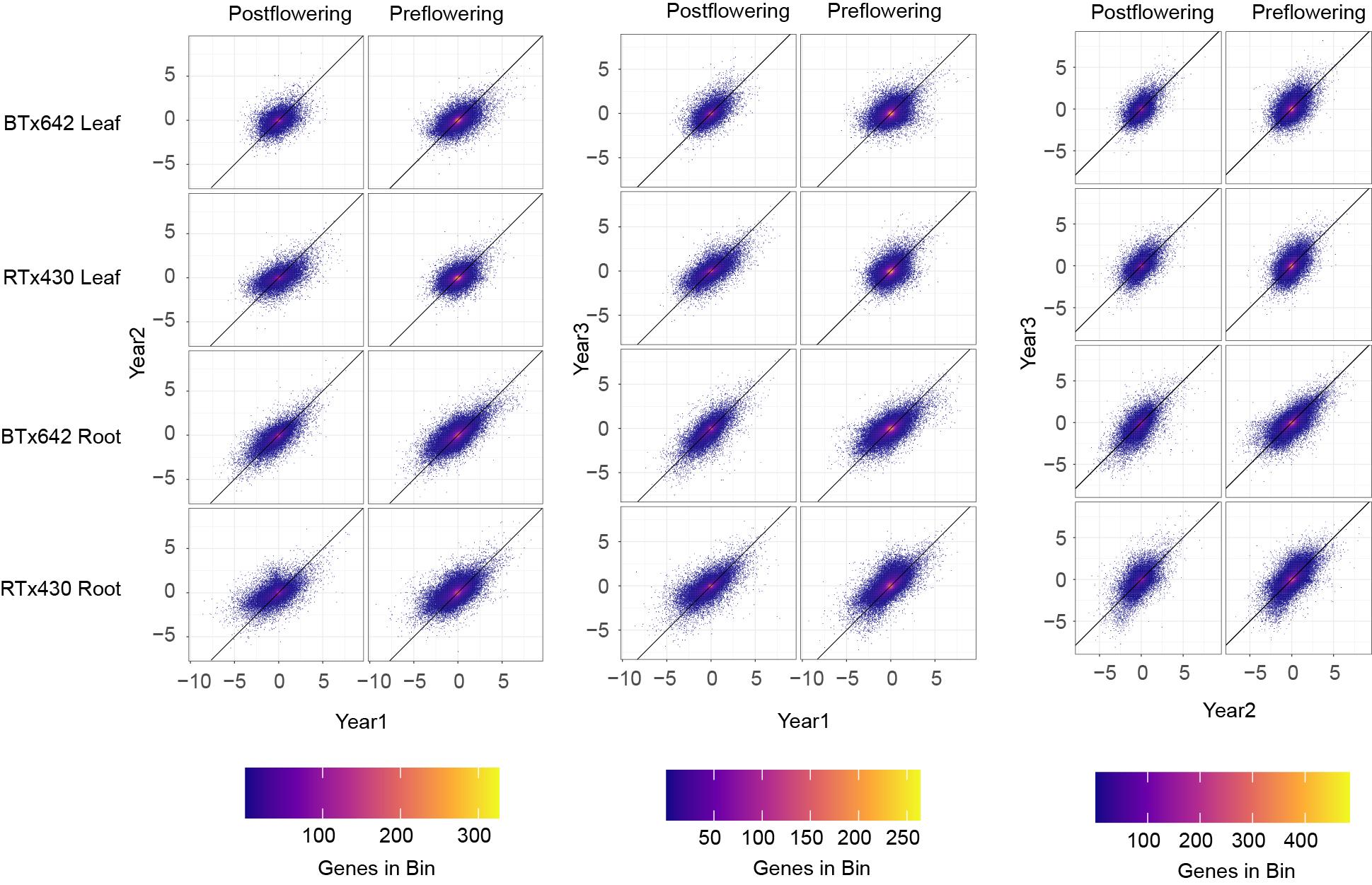** |
| --- |
| **Figure S2: Pairwise comparison of log-fold change in gene expression by year.** Shown are log2-fold changes of each gene in pairwise comparisons across years. Left, Year1 vs. Year2; middle, Year1 vs. Year3; Right, Year2 vs. Year3. Color scale represents point density (2-dimensional binning). |

| **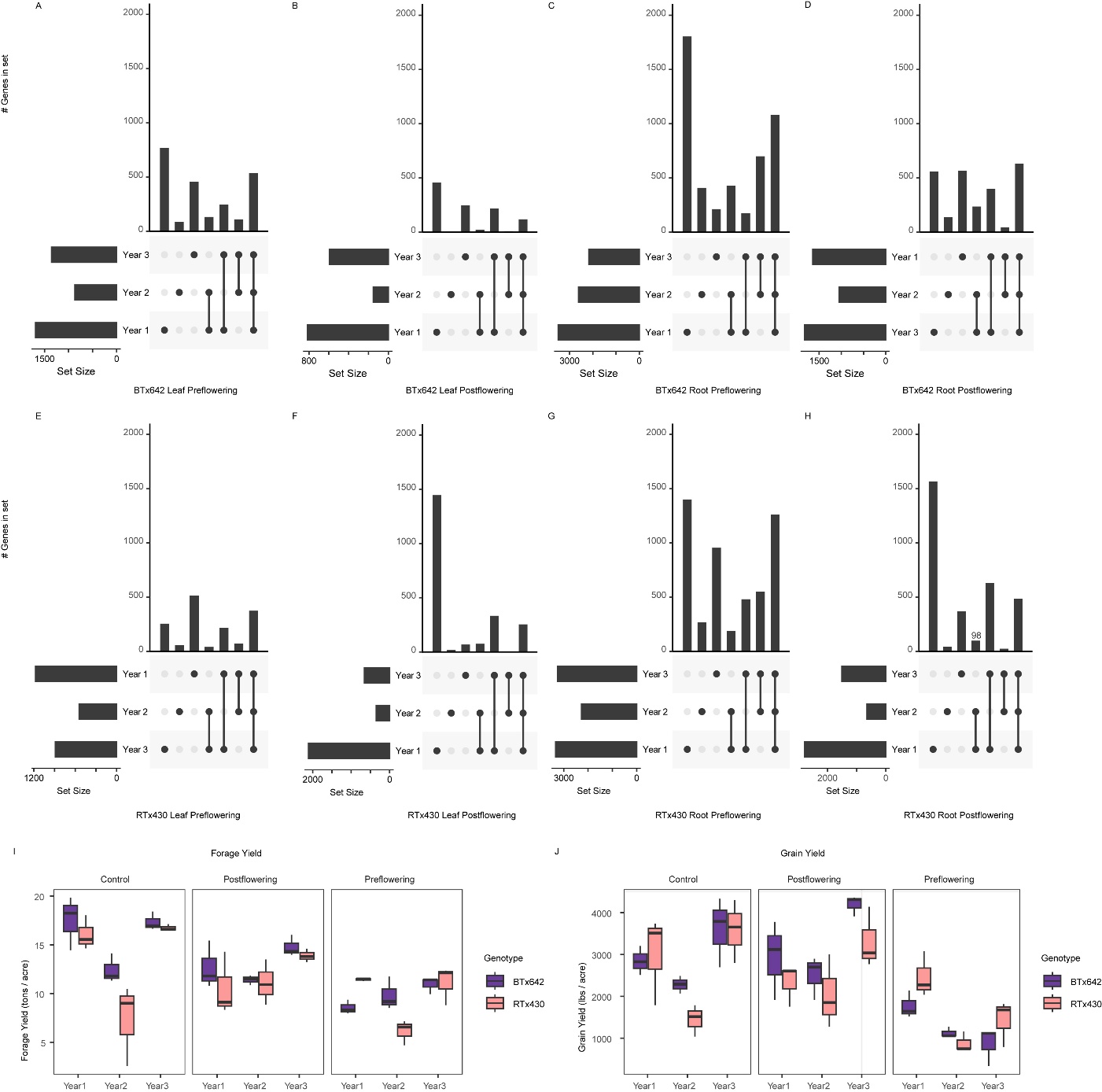** |
| --- |
| **Figure S3**: A-H) DE genes recovered from each condition per year. Shown are UpSet plots describing set intersections of DE genes identified for each condition/year combination. Individual dots represent DE genes unique to a particular year. Connected dots represent DE genes that are common to specified years. This is an expanded set of comparisons, similar to **Fig. 3**. I) Forage and J) grain yield for control, pre-flowering and post-flowering drought conditions for both genotypes across each year. |

| **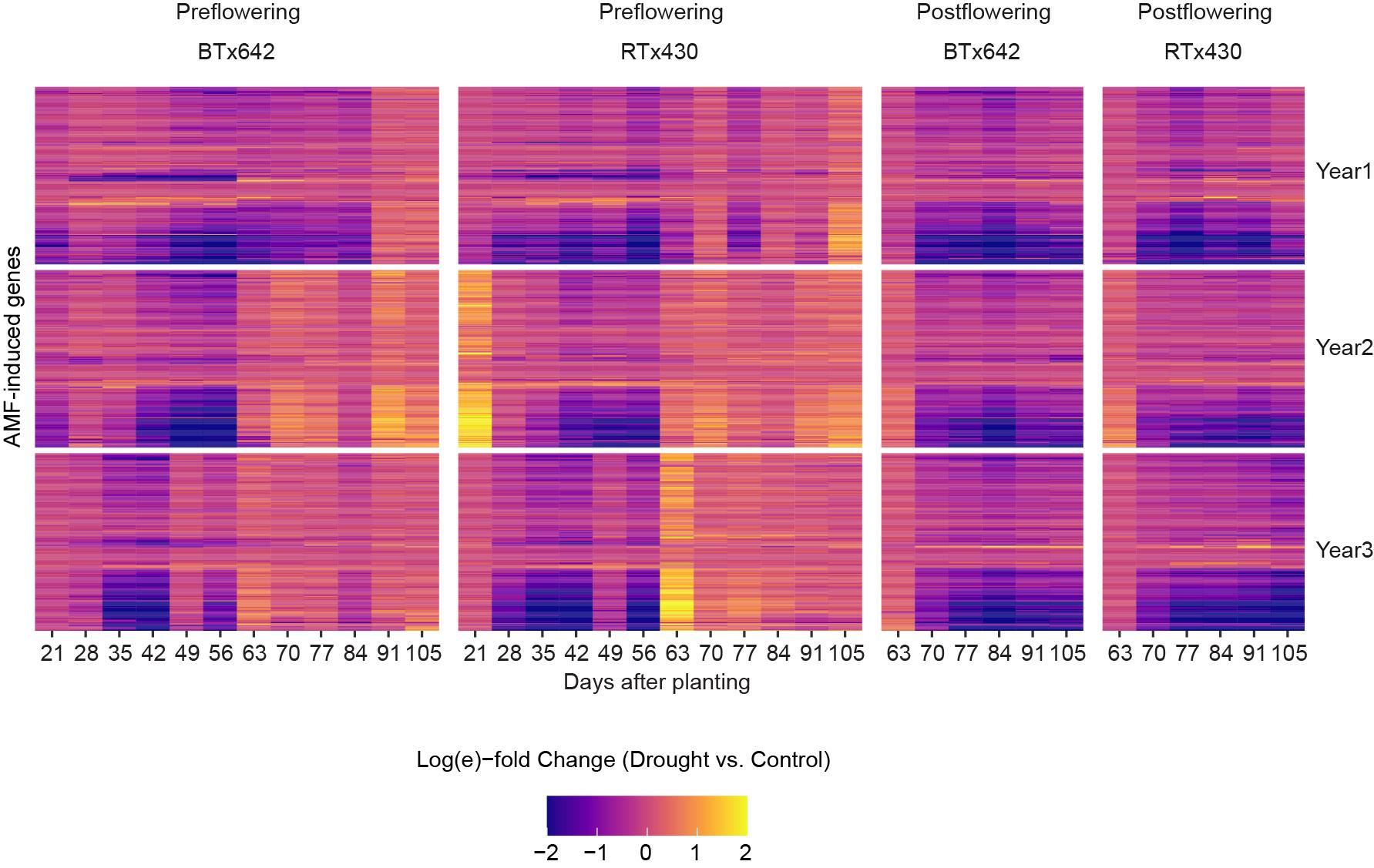** |
| --- |
| **Figure S4**: Root expression of AMF-related genes decreases during drought. Genes known to be induced by AMF are sharply down-regulated during both pre- and post-flowering drought stress in sorghum roots. Shown is a heat map of log-fold change expression across a time course for all three sampling years. |

| **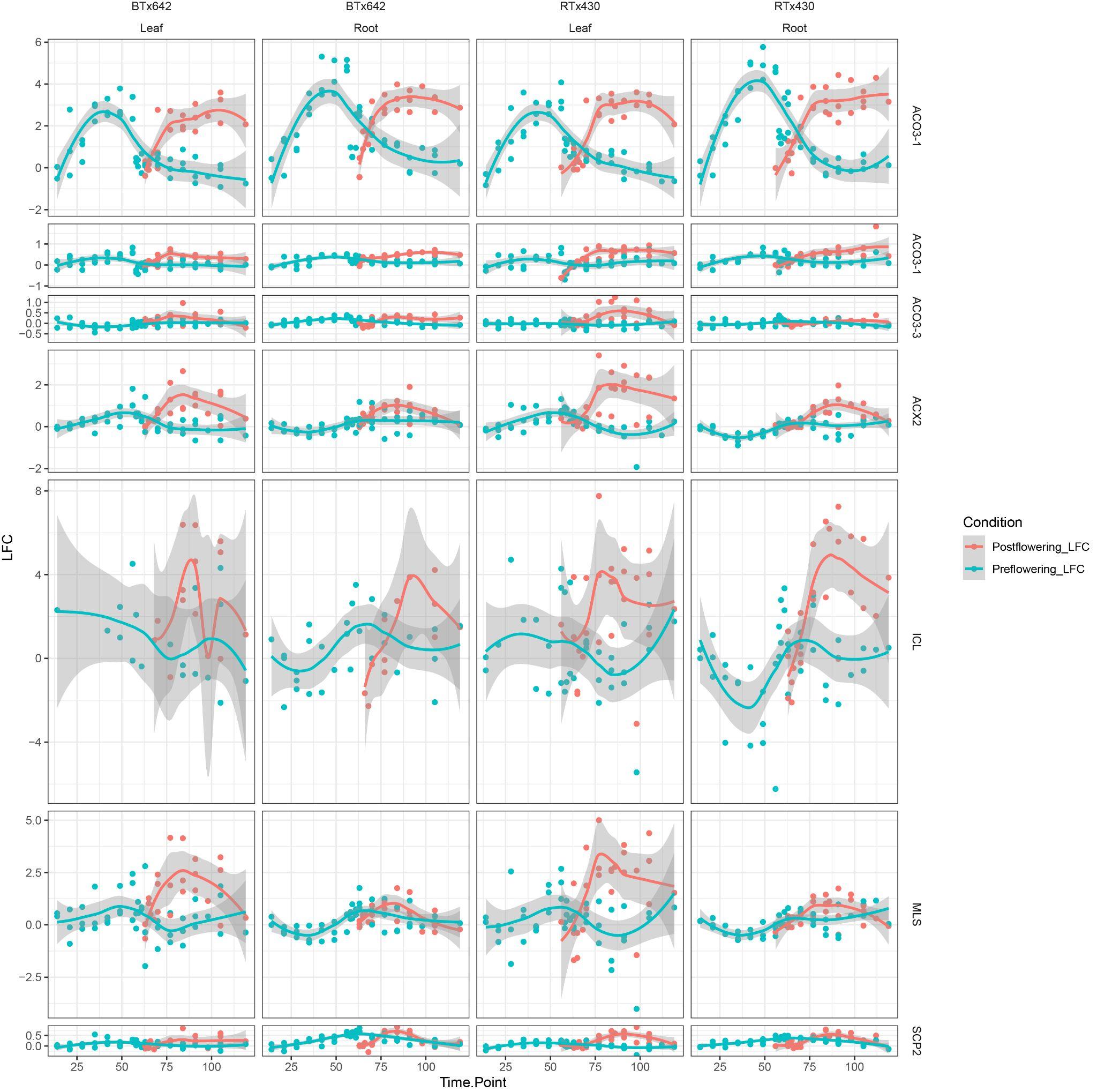** |
| --- |
| **Figure S5**: Expression of 7 pangenes corresponding to glyoxylate pathway loci in sorghum. Most genes are up-regulated in post-flowering drought stress, though several are also up-regulated during pre-flowering drought. |

| **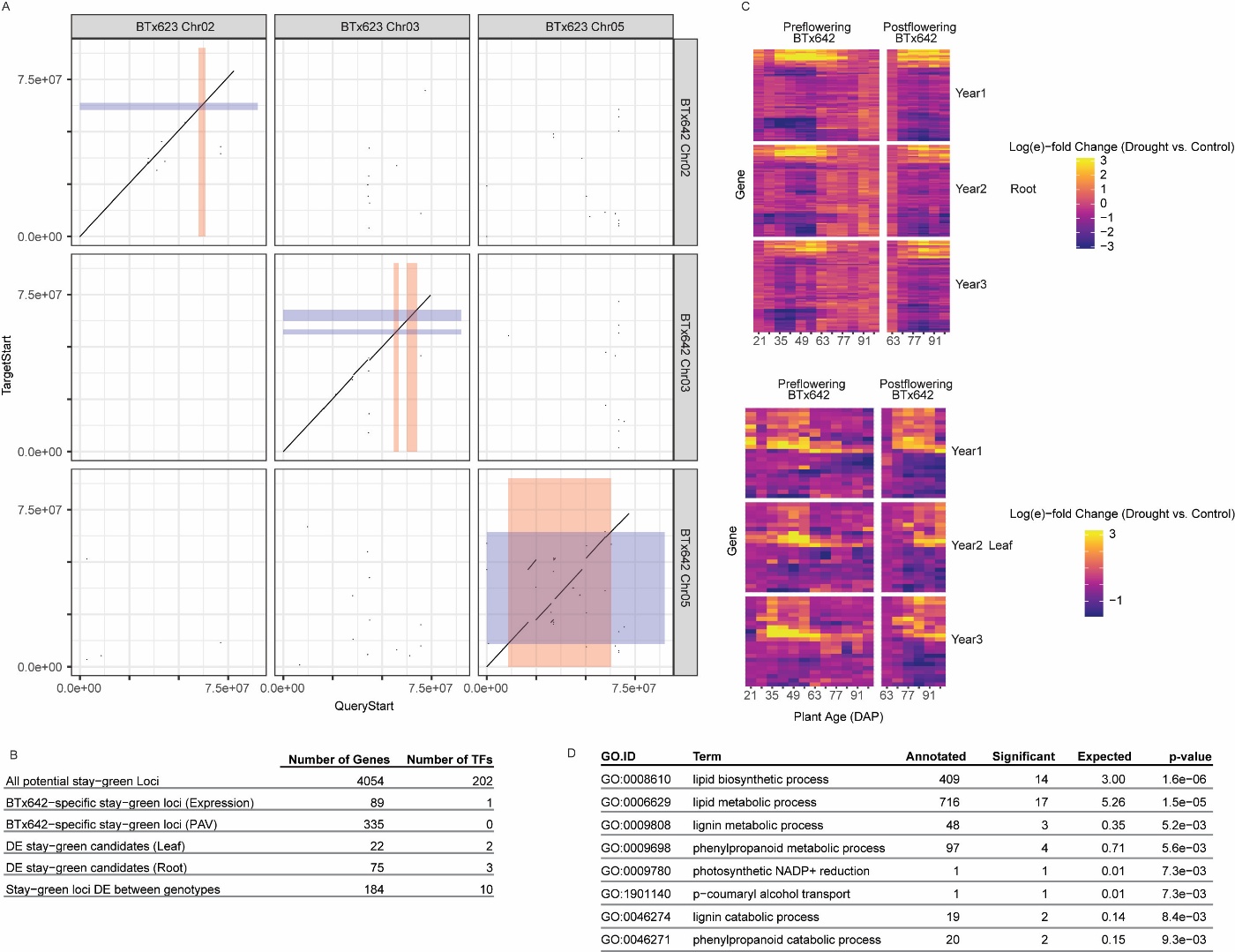** |
| --- |
| **Figure S6**: Identification of stay-green candidates in EPICON data. Potential stay-green loci were first identified by comparing the BTx623 genome to BTx642, then using these alignments to map coordinates of 4 major QTL onto the BTx642 genome (A). Genes existing within these coordinates were then further characterized as having presence-absence variation (PAV) between the RTx430 and BTx642 genomes, being exclusively expressed in the BTx642 genome, being DE between genomes, and being DE in drought in either root or leaf in BTx642. The “Total stay-green candidates” were the union set of those Stay-Green associated genes that had PAV or specific expression, or that were significantly DE between genotypes (B). Expression profiles for these genes are plotted in (C) for those DE in Root or Leaf. GO term enrichment was also performed for these genes (D). |

| **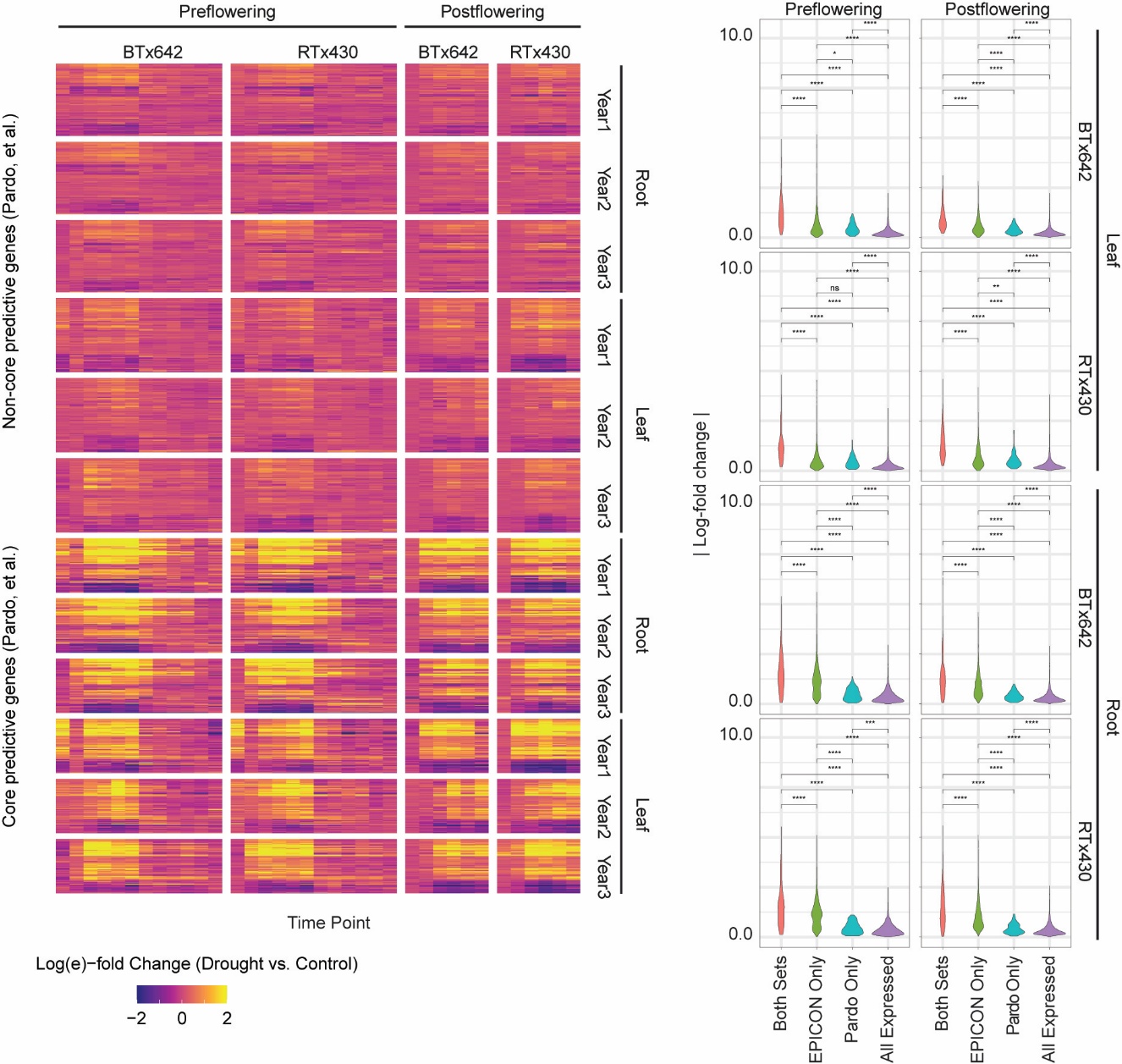** |
| --- |
| **Figure S7: Comparison to meta-analysis of sorghum drought transcriptomes.** Core drought genes were compared to genes identified as important for drought (Pardo, et al., 2023). The left panel shows a heatmap of expression of genes identified by Pardo, et al. that were either identified or not as Core drought genes in the present study. The right panel shows a violin plot of expression for the Core drought and Pardo predictive gene sets, compared to the set of all expressed genes. Asterisks indicate significance levels for pairwise Wilcoxon Rank Sum tests (p < 0.05*; p < 0.01**; p < 0.001***; p < 0.0001****) |

| **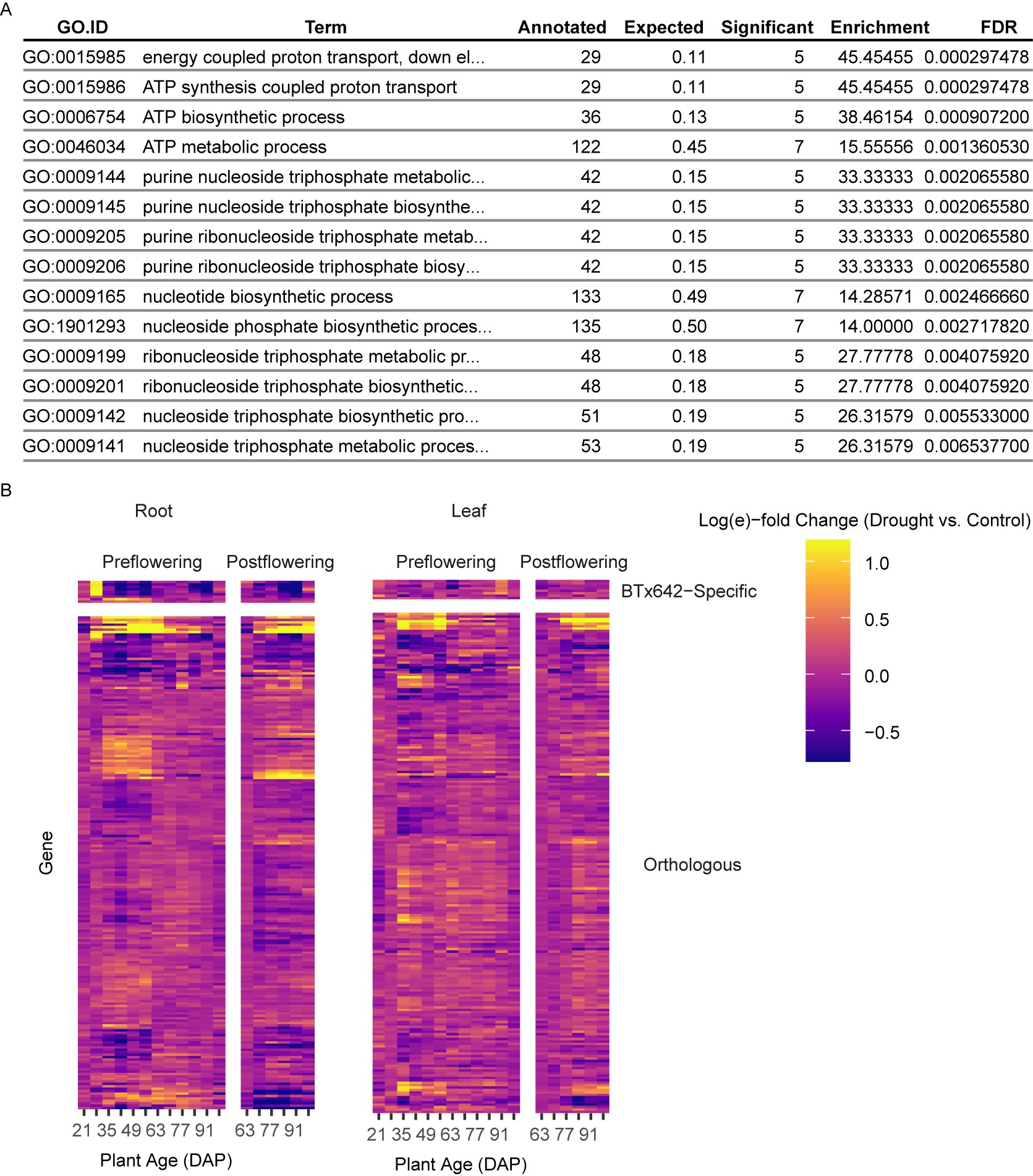** |
| --- |
| **Figure S8: GO-enrichment analysis of BTx642-specific genes**. **A**) We identified 2,748 BTx642 loci that did not have an ortholog in RTx430, and performed a GO enrichment analysis. **B)** We further identified the genes associated with significant GO terms and their offspring and plotted their expression profile across pre-and post-flowering drought in leaf and root tissue. 9 genes associated with this expanded set of GO terms were BTx642-specific. |
